# Lactylation in liver fibrosis: from molecular mechanisms to targeted therapeutic strategies

**DOI:** 10.3389/fphar.2026.1930280

**Published:** 2026-09-28

**Authors:** Zhiwei Dong, Feibo Xu, Mei Ye, Lu Wang

**Affiliations:** 1 Department of Thyroid and Breast Surgery, Yantai Affiliated Hospital of Shandong Medical and Pharmaceutical University, Yantai, Shandong, China; 2 Department of Histology and Embryology, School of Basic Medicine, Shandong Medical and Pharmaceutical University, Yantai, Shandong, China; 3 Department of Rehabilitation Medicine, Hangzhou First Peoples’ Hospital Xiasha Campus, Hangzhou Rehabilitation Hospital, Hangzhou, Zhejiang, China; 4 Department of Diagnostics, Second School of Clinical Medicine, Shandong Medical and Pharmaceutical University, Yantai, Shandong, China

**Keywords:** hepatic stellate cells, lactylation, liver fibrosis, post-translational modification, targeted therapy

## Abstract

Metabolic reprogramming and epigenetic remodeling of hepatic stellate cells (HSCs) represent central driving events in liver fibrosis. Lactylation, a lactate-mediated post-translational modification (PTM), is proposed to converge on histone H3 lysine 18 lactylation (H3K18La) through the HK2-KAT8 positive feedback loop, the IGF2BP2-N6-methyladenosine (m6A)-aldolase A (ALDOA) cascade, and the HKDC1-ORMDL3 pathway, potentially driving pro-fibrotic transcriptional programs in a synergistic manner. H3K18La does not operate in isolation; its site-specific competition with acetylation, cascade amplification with m6A methylation, cooperation with G9a/DNMT1-mediated methylation, and antagonism with ubiquitination form a PTM interaction network. Therapeutically, interventions targeting lactate-metabolizing enzymes (HK2, LDH), lactylation writers (KAT8), and specific lactylation sites (PGK1-K353 interfering peptides) have demonstrated anti-fibrotic potential in preclinical models. Natural products including curcumol, limonin, and salidroside exhibit multi-target regulatory advantages. Muscle-derived SORBS3 lactylation-mediated trans-organ signaling and lactylation gene-based molecular subtyping expand the systemic dimensions of lactylation. This narrative review proposes a working hypothesis positioning H3K18La as a metabolic-epigenetic convergence node and outlines future directions including site-specific interventions, cell type-resolved lactylomics, and molecular subtyping-driven clinical trials, providing an integrated framework from basic research to translational applications for liver fibrosis.

## 1 Introduction

Lactate, the end product of glycolysis, was long regarded as a metabolic waste product. However, this view has since been revised: lactate acts as an energy substrate and signaling molecule (Rabinowitz and Enerbäck, 2020; Wang et al., 2024a) as well as a substrate for histone lysine lactylation (Kla) (Zhang et al., 2019; Sheng et al., 2026), thereby establishing lactate as a biological bridge linking metabolism to epigenetics (Sheng et al., 2026; Qiu et al., 2026). Kla has since been identified on both histone and non-histone proteins, constituting a vast lactylome (Liu et al., 2025a; Hong et al., 2023). The liver, as the central organ of lactate metabolism, clears lactate via gluconeogenesis at the highest uptake rate among all organs (Rabinowitz and Enerbäck, 2020). Aberrant lactate metabolism is closely associated with the progress of liver disease, and lactylation is now a central topic in liver disease research (Xu et al., 2025).

Liver fibrosis represents a critical pathological stage in the progression of chronic liver disease to cirrhosis and hepatocellular carcinoma, affecting hundreds of millions worldwide (GBD, 2019 Diseases and Injuries Collaborators, 2020; Huang et al., 2023). Metabolic reprogramming, particularly enhanced aerobic glycolysis (the Warburg effect), characterizes HSC activation and immune cell functional remodeling (Trivedi et al., 2021; Horn and Tacke, 2024), with intrahepatic lactate accumulation providing abundant substrate for lactylation. Accumulating evidence demonstrates that upstream regulators including hexokinase 2(HK2), hexokinase domain containing 1(HKDC1), and IGF2BP2 enhance glycolysis through distinct metabolic pathways, converging on H3K18La and potentially activating pro-fibrotic genes such as *SOX9, ORMDL3*, and *COL1A1* (Wu et al., 2024; Han et al., 2025a). In the immune microenvironment, the H3K18La-interferon regulatory factor 4 (IRF4) axis drives Th17 differentiation to exacerbate HSC activation (Zhang et al., 2026a), while PGK1-K353 lactylation in fibroblast-specific protein 1 (FSP1)-positive macrophages sustains a pro-fibrotic milieu through a positive feedback loop (Wang et al., 2026a). Recent studies have uncovered trans-organ lactate signaling (the SORBS3-lactosome axis (Liu et al., 2025b)), exosome-lactate-lactylation metabolic circuits (Chen et al., 2026), and regulation of both histone and non-histone lactylation (Chen et al., 2025a).

These findings, however, remain fragmented: do multiple upstream pathways converge on a common epigenetic node? Do different lactylation sites exhibit functional specialization? Although recent reviews have summarized lactylation research in liver fibrosis (Xu et al., 2025; Wang et al., 2026a; Liang et al., 2025), the core question of how multiple independent pathways jointly regulate lactylation remains unanswered. To address this gap, this review proposes an integrative unifying hypothesis: HK2, HKDC1, and IGF2BP2 converge on H3K18La through distinct metabolic pathways to drive pro-fibrotic transcriptional programs. Based on this convergence hypothesis, this narrative review examines the enzymatic regulatory network of lactylation, PTM crosstalk, and immune-metabolic mechanisms. Regarding therapeutic strategies, interventions targeting lactate-metabolizing enzymes, lactylation writers, and natural products have shown significant progress in preclinical models (Guo et al., 2026; Lan et al., 2026), moving these approaches from mechanistic understanding toward clinical translation. This review also proposes individualized intervention strategies based on patient lactate metabolic phenotypes and highlights the independent functions of histone H4 lysine 12 lactylation (H4K12La), histone H4 lysine 16 lactylation (H4K16La), and histone H3 lysine 9 lactylation (H3K9La) in specific liver disease subtypes, providing conceptual support for a hierarchical regulatory model of core node dominance with multi-site coordination.

## 2 Molecular mechanisms and regulatory network of lactylation

### 2.1 Lactate transporters: gateway regulation of lactylation substrate supply

As a core bridge linking cellular metabolism to epigenetic regulation, the function of lactylation relies on a precise regulatory network comprising three hierarchical layers: substrate supply, modification catalysis, and signal recognition (Xu et al., 2024) (Table 1). The intracellular accumulation of lactate, the substrate for lactylation, depends on both glycolytic rate and the precise regulation of plasma membrane monocarboxylate transporters (MCTs) (Halestrap, 2012; Felmlee et al., 2020). Among the MCT family, MCT1 (SLC16A1) and MCT4 (SLC16A3) are the primary carriers for lactate transmembrane transport, whose differential expression and directional transport determine lactate compartmentalization between intracellular and extracellular spaces and across cell types. In the liver fibrosis microenvironment, dysregulated MCT expression directly affects lactylation substrate availability. Evidence indicates that MCT1 expression in HSCs is significantly upregulated during fibrogenesis, promoting lactate uptake to enhance collagen I expression and HSC activation; *MCT1* inhibition or knockout effectively attenuates liver fibrosis (Min et al., 2024). MCT1 exhibits cell type-specific expression and functional heterogeneity in the liver, with distinct transport directionality in hepatocytes versus HSCs, as reviewed by Drozdzik et al. (Drozdzik et al., 2020). This cell type-specific transport directionality provides a potential window for precision intervention. MCT4 regulates the expression of key lipid metabolism genes in NAFLD (Gou et al., 2025), suggesting that MCT family functions extend well beyond lactate transport in metabolic liver diseases. However, the role of MCTs in liver fibrosis remains controversial: saikosaponin B1 alleviates hepatic fibrosis by inhibiting the lactate dehydrogenase A (LDHA)-MCT1/4 axis (Wang et al., 2026b), whereas the MCT1/4 inhibitor syrosingopine paradoxically activates HSCs and exacerbates fibrosis (Guo et al., 2024). This bidirectionality suggests that the outcomes of MCT-targeted interventions may depend on fibrosis stage and lactate subcellular compartmentalization. Emerging evidence also implicates MCT1 in the lactate shuttle between hepatocytes and HSCs, complicating therapeutic targeting.

**TABLE 1:** Regulatory enzymes and transporters of lactylation in liver fibrosis.

Type	Enzyme/Protein	Cell/Tissue	Target/Substrate	Function in liver fibrosis	Ref
Transporter					
Transporter	MCT1 (SLC16A1)	HSC/hepatocyte	Lactate transport	Promotes lactate uptake in HSC, enhances HSC activation; mediates lactate efflux in hepatocytes	(Min et al., 2024)
Transporter	MCT4 (SLC16A3)	Hepatocyte	Lactate transport + lipid metabolism	Regulates lipid metabolism genes in NAFLD; functions extend beyond lactate transport	(Gou et al., 2025)
Writer					
Writer	p300/CBP	Mammalian	H3K18 and others	First identified lactyltransferase; catalyzes histone and non-histone lactylation using lactyl-CoA	(Zhang et al., 2019)
Writer	KAT8 (MOF)	HSC	HK2-K346	Catalyzes HK2 K346 lactylation, blocks HUWE1-mediated ubiquitination, forms self-amplifying positive feedback loop	(Guo et al., 2026)
Writer/Eraser	HDAC1/2/3	HSC and other cells	H3K18/H4K5	Lactate concentration-sensitive bidirectional switch: delactylase at low lactate, lactyltransferase at high lactate	(Gonzatti et al., 2025)
Eraser					
Eraser	HDAC1/2/3	HSC and other cells	H3K18/H4K5	Primary delactylases under physiological conditions; maintain basal modification levels	(Gonzatti et al., 2025)
Eraser	SIRT1	Mammalian	Multiple proteins	NAD + -dependent delactylase	(Du et al., 2024)
Eraser	SIRT3	Mammalian	H4K16/H3K9	Prominent delactylase activity for H4K16La	(Fan et al., 2023; Du et al., 2024)
Reader					
Reader	TRIM33	Mammalian	Histone	Regulates macrophage M1/M2 polarization	(Nuñez et al., 2024)
Reader	BRD9	Human (HCC validated; HSC pending)	H3K18 (bromodomain)	Recognizes H3K18La, recruits ncBAF complex to enhancers/promoters, drives pro-fibrotic and oncogenic transcription	(Wei et al., 2026)

HDAC1/2/3 function as bidirectional switches: they act as erasers under low lactate and as writers under high lactate conditions, representing a unique lactate-sensitive regulatory mechanism.

### 2.2 Enzymatic regulation of lactylation

Lactate dehydrogenase (LDH) controls the first step of the lactylation cascade by converting pyruvate to lactate, thereby setting the amount of intracellular substrate available for modification. In metabolically active states such as HSC activation, enhanced aerobic glycolysis leads to substantial lactate accumulation, providing ample substrate for downstream lactylation (Rho et al., 2023). Lactyl-CoA serves as the direct donor for lactyl group transfer, although its nuclear origin had remained elusive. Yao et al. identified nuclear GTPSCS as a lactyl-CoA synthetase, filling this critical gap (Liu et al., 2025c). In the same year, AARS1/2 were characterized as lactyl-CoA-independent global lactyltransferases that directly utilize free L-lactate to catalyze lactylation in an ATP-dependent manner (Li et al., 2024a), revealing the diversity of lactylation substrate utilization pathways (Zhang et al., 2025).

The actual transfer of lactyl groups onto lysine residues is catalyzed by a family of enzymes known as writers. P300/CBP was the first identified lactyltransferase, utilizing lactyl-CoA to catalyze lactylation of histone and non-histone proteins (Zhang et al., 2019) (Table 1). KAT8 (also known as MOF), a MYST family acetyltransferase, has a distinct role in liver fibrosis: in activated HSCs, KAT8 specifically catalyzes HK2 lactylation at the K346 site, which protects HK2 from ubiquitin-proteasomal degradation by blocking recognition by the HUWE1 E3 ubiquitin ligase, thereby maintaining HK2 protein stability and glycolytic activity (Guo et al., 2026). Notably, the recent discovery by Gonzatti et al. has overturned the traditional writer-eraser dichotomy: Class I histone deacetylases (HDAC1/2/3) can reverse their classical deacylase activity upon elevated lactate concentrations, directly utilizing lactate (rather than lactyl-CoA) to catalyze lysine lactylation (Gonzatti et al., 2025). This finding implies that HDACs function as lactate concentration-sensitive bidirectional switches: acting as erasers under low-lactate conditions to maintain baseline modification levels, while switching to writers under high-lactate conditions to amplify lactylation signals. This mechanism may explain the sustained elevation of lactylation levels following HSC activation.

Erasers remove lactyl groups, thereby maintaining the modification in a state of dynamic equilibrium. Despite their bidirectional catalytic potential, HDAC1-3 primarily exert delactylase activity under physiological conditions (Gonzatti et al., 2025). Sirtuin 1 (SIRT1) and sirtuin 3 (SIRT3) are two other important NAD + -dependent delactylases, with SIRT3 exhibiting prominent delactylation activity toward H4K16La (Fan et al., 2023; Du et al., 2024). Readers, in turn, translate lactylation marks into downstream functional outputs through specific recognition. The bromodomain of TRIM33 was the first identified histone Kla reader, capable of binding Kla with sub-micromolar affinity and regulating macrophage M1/M2 polarization (Nuñez et al., 2024). Recently, bromodomain-containing protein 9 (BRD9) was identified as a specific reader for H3K18La; its bromodomain recognizes H3K18La through a conserved acetyl-lysine binding pocket and recruits the non-canonical BAF (ncBAF) chromatin remodeling complex to target gene enhancers and promoters, increasing chromatin accessibility to drive transcription of pro-fibrotic and oncogenic genes such as *SPARC* and *TMEM64* (Wei et al., 2026). Given that H3K18La is similarly elevated in activated HSCs, the BRD9-H3K18La-ncBAF axis warrants further investigation in liver fibrosis. In summary, writers, erasers, and readers cooperate through substrate concentration sensing, enzymatic activity switching, and chromatin remodeling to construct a complete signal transduction chain from metabolic input to transcriptional output in lactylation regulation (Sheng et al., 2026; Peng and Du, 2025).

### 2.3 Crosstalk between lactylation and other post-translational modifications

Lactylation does not operate in isolation but participates in a complex intracellular signaling network with acetylation, methylation, phosphorylation, and ubiquitination (Hu et al., 2024). These interactions occur at multiple levels: competitive occupancy of the same lysine residue, coordinated regulation of different sites on the same protein, and indirect regulation through modifier enzyme activity or substrate availability (Figure 1).

**FIGURE 1:**
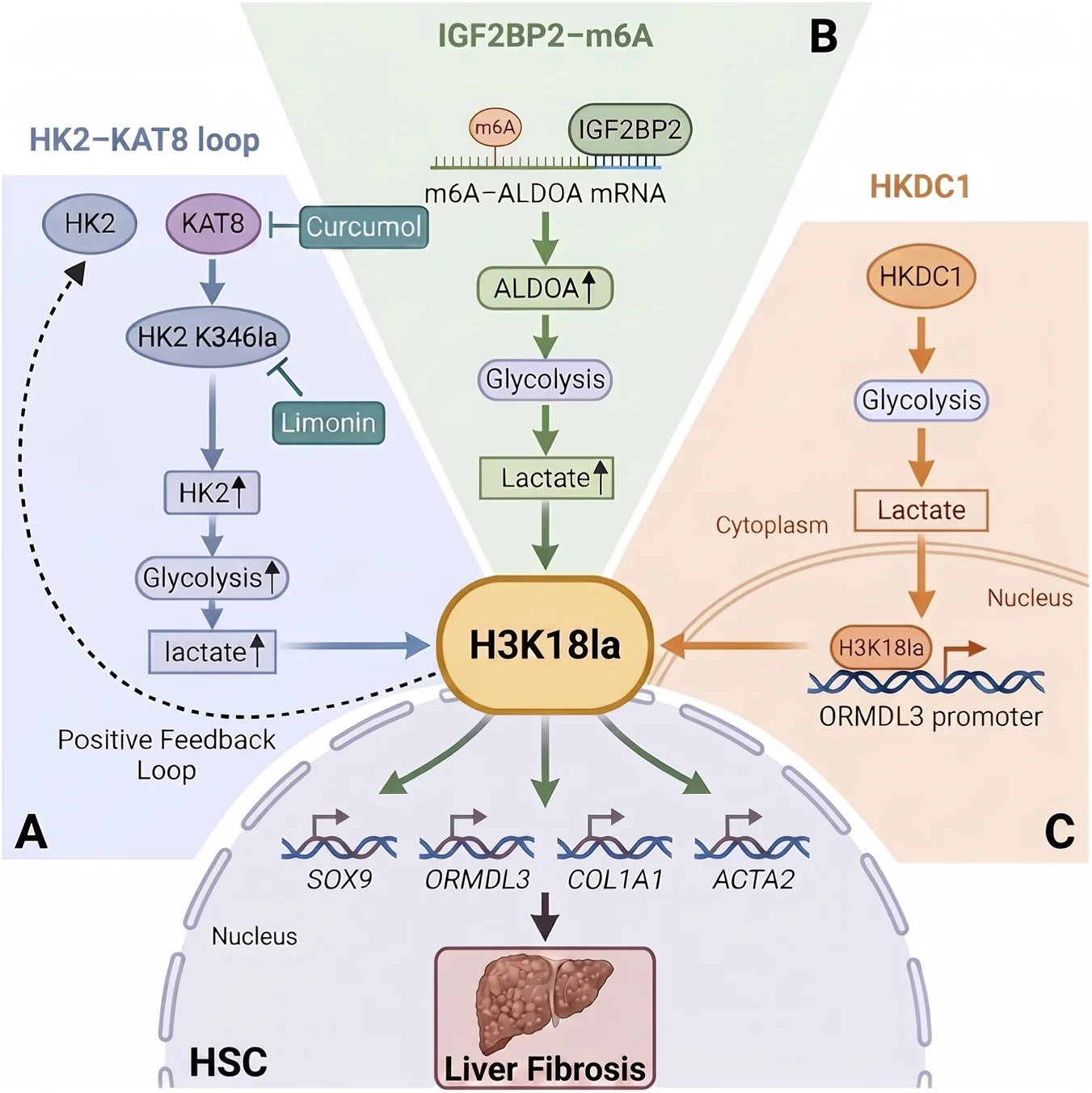
Schematic overview of H3K18la as a metabolic-epigenetic convergence hub in liver fibrosis. Three convergent axes drive H3K18la accumulation and pro-fibrogenic transcription: **(A)** the HK2-KAT8 positive feedback loop, in which HK2 K346 lactylation enhances glycolysis and lactate production; **(B)** the IGF2BP2-m6A-ALDOA axis, whereby m6A-modified ALDOA mRNA promotes glycolytic flux; and **(C)** the HKDC1-cytoplasmic lactate/nuclear H3K18la-ORMDL3 cascade, which activates SOX9, ORMDL3, COL1A1, and ACTA2 expression. Curcumol and limonin are indicated as candidate modulators. Abbreviations: ACTA2, actin alpha 2; ALDOA, aldolase A; COL1A1, collagen type I alpha 1 chain; H3K18la, histone H3 lysine 18 lactylation; HK2, hexokinase 2; HKDC1, hexokinase domain-containing protein 1; HSC, hepatic stellate cell; IGF2BP2, insulin-like growth factor 2 mRNA-binding protein 2; KAT8, lysine acetyltransferase 8; m6A, N6-methyladenosine; ORMDL3, ORMDL sphingolipid biosynthesis regulator 3; SOX9, SRY-box transcription factor 9.

The direct competition between lactylation and acetylation at the H3K18 site represents the most typical example of PTM interplay in liver fibrosis (Rho et al., 2023). Both modifications share H3K18 as the substrate residue, and only one acylation mark can occupy this site at any given time. CUT&Tag analysis revealed that in activated HSCs, H3K18La occupancy at pro-fibrotic gene promoters reaches 42.5%, compared to only 27.4% for histone H3 lysine 18 acetylation (H3K18ac). Functionally, transforming growth factor-β1 (TGF-β1) stimulation of Lx-2 cells leads to sustained H3K18La elevation and progressive H3K18ac decline, and lactate-driven α-SMA expression can be completely reversed by exogenous acetate. Class I HDAC inhibitors apicidin and MS275 competitively displace H3K18La by upregulating H3K18ac, effectively inhibiting HSC activation gene expression, providing direct pharmacological evidence for targeting the lactylation-acetylation balance. Non-histone lactylation is also globally elevated while acetylation is concomitantly decreased in activated HSCs, suggesting that this competitive relationship is not limited to histones but reflects a systematic shift in the lactylation-acetylation balance in liver fibrosis (Rho et al., 2023).

The crosstalk between lactylation and N6-methyladenosine (m6A) RNA methylation as well as ubiquitination represents two additional important PTM coordination modes. The m6A reader protein IGF2BP2, highly expressed in activated HSCs, stabilizes the mRNA of the glycolytic enzyme *ALDOA* in an m6A-dependent manner, enhancing glycolytic flux and lactate production. Accumulated lactate drives H3K18La elevation, which further activates pro-fibrotic gene transcription, forming an IGF2BP2-m6A-ALDOA-lactate-H3K18La cascade amplification loop (Zhou et al., 2024). The core feature of this pathway is that IGF2BP2 does not directly regulate lactylation enzymes but indirectly amplifies lactylation signals by enhancing glycolytic metabolic flux. This mechanism couples RNA epitranscriptomics with histone lactylation, revealing a multi-layered synergistic regulatory network spanning RNA and chromatin. In contrast to cascade amplification, lactylation and ubiquitination engage in direct steric competition: when a lysine residue is occupied by lactylation, ubiquitination cannot be installed. The prototypical example is KAT8-catalyzed HK2 K346 lactylation, which physically blocks recognition by the HUWE1 E3 ubiquitin ligase, protecting HK2 from K48-linked ubiquitin-proteasomal degradation and maintaining HK2 protein stability and HSC glycolytic activity (Guo et al., 2026). This lactylation-ubiquitination competitive occupancy mode likely exists broadly within the metabolic-epigenetic regulatory network of liver fibrosis, providing a new molecular framework for understanding the aberrant stability of metabolic enzymes in the fibrotic microenvironment.

Lactylation also engages histone and DNA methylation in a cooperative circuit at the chromatin level. G9a-catalyzed H3K9me2 and DNA methyltransferase (DNMT)-mediated DNA methylation epigenetically silence gluconeogenic genes, relieving suppression of the glycolytic phenotype. The resulting lactate accumulation provides metabolic substrate for H3K18La enrichment at pro-fibrotic gene promoters, while H3K18La transcriptionally activates glycolytic genes, forming a feedforward amplification loop linking lactylation, glycolysis, lactate production, and further lactylation (Barcena-Varela et al., 2021). The G9a/DNMT dual inhibitor CM272 can intercept this cooperation at the methylation source, indirectly suppressing histone lactylation levels. Extensive crosstalk between lactylation and phosphorylation has been reported in other disease models: the GCN5-ERK lactylation-phosphorylation positive feedback loop amplifies lactate-driven tumor progression (Huang et al., 2026), and lactylation of Ku70 and Ku80 in lung cancer cells is closely associated with NHEJ repair efficiency (He et al., 2025a). However, whether these crosstalk mechanisms operate in liver fibrosis remains to be determined.

The site-specific competition between lactylation and acetylation, the metabolic-epigenetic cooperation with methylation, the protein stability antagonism with ubiquitination, and the amplification with m6A methylation collectively constitute a PTM interaction network centered on H3K18La. In liver fibrosis, this network integrates metabolic signals with epigenetic outputs, influencing the trajectory of HSC activation and fibrotic progression.

## 3 Mechanisms of lactylation in liver fibrosis

### 3.1 HSC activation and the metabolism-lactylation axis

HSC activation is the central cellular event in liver fibrogenesis, accompanied by significant metabolic remodeling: a shift from oxidative phosphorylation-dominated metabolism in the quiescent state to aerobic glycolysis as the primary metabolic mode (Trivedi et al., 2021). In the liver injury microenvironment, multiple signaling pathways coordinately drive this transition. Canonical Wnt signaling directly enhances HSC glycolytic flux through the LDH-A/hypoxia-inducible factor-1α (HIF-1α) transcriptional complex, providing a key transcriptional driving force for metabolic reprogramming (Wang et al., 2024b). TGF-β1, the most potent pro-fibrotic factor, significantly upregulates glucose transporter GLUT1 expression through both Smad-dependent canonical and p38 MAPK/PI3K-AKT non-canonical pathways, promoting glucose uptake and aerobic glycolysis (Zhou et al., 2021). The PNPLA3 I148 M variant enhances HSC glycolysis by activating Hedgehog and Yap signaling pathways, resulting in increased lactate release and inhibited AMPK phosphorylation, further exacerbating pro-inflammatory and pro-fibrotic phenotypes (Bruschi et al., 2020). These findings collectively demonstrate that metabolic reprogramming, particularly enhanced glycolysis, is a driving factor rather than a passive consequence of HSC activation, providing a mechanistic explanation for the upstream metabolic basis of lactylation.

Recent studies have broadened our understanding of HSC metabolic plasticity and its lactylation coupling. Downregulation of phosphoenolpyruvate carboxykinase one drives HSC metabolic flux toward glycolysis by relieving the antagonism of gluconeogenesis on glycolysis, thereby promoting liver fibrosis (Novoa et al., 2026). Activated HSCs release glycolysis-dependent extracellular vesicles carrying pro-fibrotic factors to coordinate intrahepatic intercellular communication, amplifying paracrine propagation of fibrotic signals (Khanal et al., 2024). Furthermore, glutamine metabolism provides key anaplerotic fuel for HSC proliferation and activation, while fatty acid oxidation remodeling indirectly regulates the histone acetylation-lactylation balance through effects on the acetyl-CoA pool, broadening HSC metabolic adaptation (Trivedi et al., 2021). These multi-layered metabolic adaptations constitute the metabolic foundation for lactylation substrate supply during HSC activation and highlight the potential of metabolic nodes as anti-fibrotic intervention targets.

### 3.2 Core signaling pathways of lactylation-driven HSC activation

In activated HSCs, HK2, as the first rate-limiting enzyme of glycolysis, is central to metabolic-epigenetic regulation (He et al., 2025a). HK2 induction is a prerequisite for H3K18La, rather than H3K18ac, to drive pro-fibrotic gene transcription; *HK2* knockout significantly reduces H3K18La levels and inhibits HSC activation (Rho et al., 2023). Integrative multi-omics analysis has confirmed the significant upregulation of HK2 in HSCs and its close association with fibrosis progression (Han et al., 2025a). Post-translationally, HK2 protein stability is governed by a lactylation-dependent mechanism: lactate accumulated in activated HSCs activates the acetyltransferase KAT8, which specifically catalyzes HK2 K346 lactylation (Figure 2). This modification blocks recognition by the HUWE1 E3 ubiquitin ligase through steric hindrance, protecting HK2 from K48-linked ubiquitin-proteasomal degradation, thereby forming a self-amplifying positive feedback loop of lactate-driven KAT8-mediated HK2 lactylation, HK2 protein stabilization, sustained glycolysis enhancement, and further lactate accumulation (Guo et al., 2026). The natural compound curcumol directly binds to the TYR361 and THR319 residues of KAT8 to inhibit its catalytic activity, interrupting this loop and inducing necroptosis of activated HSCs (Guo et al., 2026). Limonin, as a novel HK2 allosteric inhibitor, directly suppresses HK2 enzymatic activity and histone lactylation, exhibiting anti-fibrotic properties *in vivo* (Lan et al., 2026). These two classes of drugs target the HK2-H3K18La axis at the level of the lactylation writer and the metabolic enzyme, respectively, providing complementary pharmacological strategies for intervening in lactylation-driven HSC activation.

**FIGURE 2:**
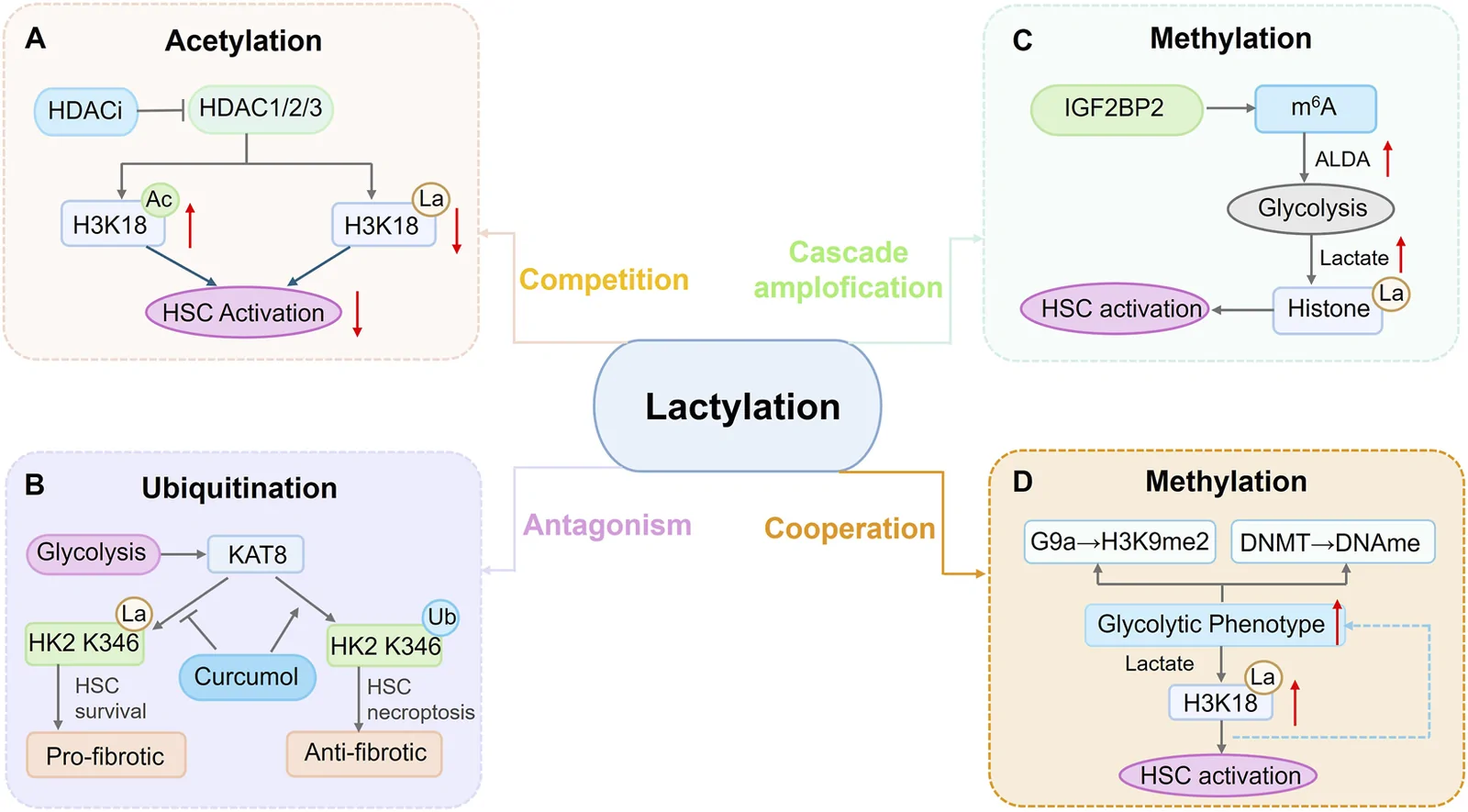
Post-translational modification crosstalk network centered on lactylation in hepatic stellate cell activation. Four distinct interaction modalities converge on histone and non-histone lactylation: **(A)** H3K18la–H3K18ac competition at the same lysine residue, with HDAC inhibitors displacing lactylation to suppress activation. **(B)** m6A–IGF2BP2 cascade amplification of glycolysis, providing lactate substrate for histone lactylation via ALDOA mRNA stabilization. **(C)** HK2 K346 lactylation–ubiquitination antagonism, where lactylation blocks HUWE1-mediated proteasomal degradation; Curcumol targets KAT8 to tip this balance. (D) G9a/DNMT methylation cooperation with lactylation by enhancing glycolysis to supply lactate substrate, which in turn reinforces glycolysis, establishing a self-amplifying circuit. Abbreviations: Ac, acetylation; ALDOA, aldolase A; DNAm, DNA methylation; DNMT, DNA methyltransferase; G9a, histone-lysine N-methyltransferase 2A; H3K9me2, histone H3 lysine 9 dimethylation; H3K18la, histone H3 lysine 18 lactylation; HSC, hepatic stellate cell; HDAC, histone deacetylase; HDACi, histone deacetylase inhibitor; IGF2BP2, insulin-like growth factor 2 mRNA-binding protein 2; KAT8, lysine acetyltransferase 8; La, lactylation; m6A, N6-methyladenosine; Ub, ubiquitination.

IGF2BP2, a newly identified m6A reader protein, plays a unique cascade regulatory role in liver fibrosis by bridging RNA epitranscriptomics and histone lactylation. IGF2BP2 is significantly overexpressed in fibrotic liver tissue and activated HSCs, stabilizing *ALDOA* mRNA in an m6A-dependent manner to enhance ALDOA protein expression, thereby promoting glycolytic flux and lactate production. Accumulated lactate drives H3K18La elevation, which further activates pro-fibrotic gene transcription, forming an IGF2BP2/ALDOA/lactate/H3K18La/fibrotic gene amplification loop. Loss-of-function experiments have confirmed that IGF2BP2 inhibition blocks HSC activation and attenuates liver fibrosis (Zhou et al., 2024). This discovery functionally couples m6A modification with histone lactylation, revealing a multi-layered epigenetic synergistic network spanning RNA metabolism and chromatin.

HKDC1, a homologous hexokinase of HK2, participates in HSC lactylation regulation through a signaling axis independent of HK2. HKDC1 expression is significantly upregulated in cirrhotic liver tissue and TGF-β1-activated HSCs. HKDC1 enhances glycolysis to increase intracellular lactate levels, which specifically increases H3K18La modification at the *ORMDL3* gene promoter, transcriptionally activating *ORMDL3* and driving HSC activation (Zhang et al., 2026a). *In vivo* silencing of *HKDC1* attenuates CCl4-induced mouse liver fibrosis, while *in vitro* knockdown inhibits HSC activation and glycolysis (Zhang et al., 2026a). This pathway first links HKDC1, rather than the classical HK2, with H3K18La and a specific downstream effector gene (ORMDL3), expanding the molecular network of lactylation-regulated HSC activation.

Integrating the three pathways described above, this review proposes an integrative working hypothesis (Figure 2): in the liver fibrosis microenvironment, the HK2-KAT8 positive feedback loop, the IGF2BP2-m6A-ALDOA cascade amplification loop, and the HKDC1-ORMDL3 transcriptional activation pathway enhance glycolysis and promote lactate accumulation through distinct metabolic routes, converging on H3K18La as the epigenetic node to drive pro-fibrotic transcriptional programs (SOX9, ORMDL3, COL1A1, ACTA2, etc.). The core evidence for this hypothesis is threefold: (1) all three pathways mediate their effector output through H3K18La, rather than alternative sites such as H4K12La or H3K14La; (2) the BRD9 reader, recently identified in hepatocellular carcinoma (HCC) models, possesses specific recognition capability for H3K18La, suggesting a potential effector coupling mechanism in liver fibrosis; and (3) the anti-fibrotic activities of drugs such as curcumol and limonin both correlate with H3K18La reduction. However, the independent roles of H4K12La in metabolic dysfunction-associated fatty liver disease (MAFLD), H3K9La in intestinal fibrosis, and H4K16La in metabolic liver disease suggest that a multi-node coordination model is an equally plausible alternative hypothesis.

A related question concerns the three pathways themselves: their spatial, temporal, and interactive relationships remain largely unresolved, and these relationships determine the scope of the hypothesis. In terms of cell identity, HSCs are heterogeneous and comprise multiple functionally distinct subpopulations (Geng and Schwabe, 2025). Whether the three pathways are co-activated within the same HSC subpopulation or segregated across distinct subpopulations has not been resolved at single-cell resolution; this distinction matters because the former would indicate intracellular synergistic convergence, whereas the latter would imply population-level functional division. Temporally, the available evidence suggests a possible temporal sequence that is also microenvironment dependent. HKDC1 is upregulated in TGF-beta1-stimulated HSCs, placing it among the early activation responses (Zhang et al., 2026a), whereas the HK2-KAT8 loop is a self-amplifying feedback circuit that may gain dominance as lactate accumulates during fibrogenesis (Guo et al., 2026). This proposed temporal switch, however, remains speculative in the absence of longitudinal studies. How these pathways interact is also unresolved. HKDC1 is known to act synergistically with other glycolytic enzymes such as FBP2 (Yu et al., 2025), and, as a hexokinase homologue of HK2, it may either compensate for HK2 loss or compete for shared glycolytic flux; neither possibility has been tested directly in liver fibrosis. These questions call for single-cell multi-omics combined with pathway-specific perturbation and stage-resolved analyses, and until they are answered the convergence hypothesis should be interpreted with caution regarding its current scope.

### 3.3 Immune cell lactylation and its regulation of liver fibrosis

Immune cell metabolic reprogramming and functional remodeling are core driving events in liver fibrosis progression (Horn and Tacke, 2024). Lactate, as the terminal product of glycolysis, reshapes the transcriptional landscape and effector functions of hepatic immune cells through histone and non-histone lactylation.

Arsenic-induced liver fibrosis models illustrate how lactylation shapes adaptive immunity: hepatocyte-derived lactate drives H3K18La-mediated upregulation of IRF4, which promotes CD4^+^ T cell differentiation into Th17 subsets and IL-17 A secretion, exacerbating HSC activation. The LDH inhibitor oxamate effectively suppresses this process and attenuates fibrosis (Chen et al., 2025b). In tumor immunity, glycolysis-derived lactate in pancreatic cancer similarly upregulates the chemokine CXCL1 through PCAF-catalyzed H3K18La, recruiting neutrophils to form an immunosuppressive microenvironment (Zhang et al., 2026b). This suggests that H3K18La-driven immune remodeling mechanisms may be conserved across diseases, although this remains to be validated in liver fibrosis models.

Kupffer cell lactylation provides a parallel example within the innate immune system, mediating complex metabolic-immune crosstalk (Guo et al., 2025; Shu et al., 2025). Global lactylome analysis of Kupffer cells identified 289 lactylation sites across 181 proteins, among which high mobility group box 1 (HMGB1) K82 lactylation is closely associated with neutrophil extracellular trap formation pathways (Sung et al., 2023). Histone lactylation also regulates macrophage inflammatory gene expression, driving the polarization switch from pro-inflammatory M1 to reparative M2 phenotype, with the TRIM33 bromodomain serving as a critical histone Kla reader in this process (Nuñez et al., 2024). In the liver fibrosis microenvironment, sustained activation of M2 macrophages is an important factor promoting HSC activation and ECM deposition. Additionally, microplastic-induced immunotoxicity models have revealed that H3K18La promotes duox expression to enhance reactive oxygen species (ROS) generation, which in turn amplifies lactylation levels, forming a positive feedback loop driving inflammation (Wang et al., 2025). Similar ROS-lactylation positive feedback mechanisms may also exist in activated macrophages during liver fibrosis, warranting in-depth investigation.

In summary, targeting lactylation pathways in specific immune cell subsets, such as inhibiting H3K18La-mediated Th17 differentiation or interfering with TRIM33-dependent macrophage M2 polarization, offers immuno-metabolic intervention strategies for liver fibrosis.

## 4 Extension of lactylation in liver fibrosis-associated liver diseases

### 4.1 Lactylation across the metabolic liver disease spectrum

Lactylation not only directly drives liver fibrosis but also broadly participates in other liver pathological processes closely related to fibrosis, exerting continuous pathogenic effects throughout the hepatitis-fibrosis-cirrhosis-hepatocellular carcinoma disease spectrum. In MAFLD, polycomb repressive complex 1 (PRC1) enhances glycolysis to drive H4K12La elevation, forming a PRC1-glycolysis-H4K12La positive feedback loop that accelerates MAFLD progression to fibrosis (Zou et al., 2026). H4K16La has been implicated in metabolic reprogramming in liver metabolic disorders, expanding the functional diversity of lactylation sites (Jiao et al., 2026). In alcohol-associated liver disease (ALD), chronic ethanol intake upregulates hepatic lactate levels, driving hepatocyte injury and fibrosis-related gene expression through H3K18La (Singh et al., 2025). Salvianic acid A alleviates alcoholic liver injury by inhibiting the bromodomain-containing protein 4 (BRD4)/HMGB1 pathway (Lan et al., 2020), suggesting that BRD4, as a potential H3K18La reader, may mediate the progression from ALD to fibrosis. In HCC, a potential consequence of advanced liver fibrosis, lactylation links metabolic reprogramming, epigenetic plasticity, and therapeutic vulnerability. Although these findings were established in HCC models rather than in models of fibrotic liver, they provide important conceptual parallels (Yin et al., 2025). BRD9, a newly identified H3K18La-specific reader, drives chromatin remodeling and oncogenic transcription (Wei et al., 2026). ABCF1-K430 lactylation, a non-histone modification, promotes malignant progression through transcriptional activation of the HIF1 signaling pathway (Hong et al., 2025). Linking metabolism to cell death, histone lactylation enhances ferroptosis resistance via NFS1 upregulation, driving post-microwave ablation metastasis (Huang et al., 2025). Pan-lactylome analysis of HCC has revealed lactylation-dependent metabolic adaptation mechanisms (Yang et al., 2023). Lactylation-based signatures tracing fibrotic progression to HCC have begun to inform prognostic stratification (Li et al., 2024b), and lactylation-associated resistance phenotypes have also been reported in other cancer types, such as radioresistance in lung adenocarcinoma (He et al., 2025b).

### 4.2 The muscle-liver trans-organ lactylation signaling axis

The pathogenic role of lactylation is not confined to the liver but extends to remote regulation through trans-organ signaling axes. Liu et al. first identified a muscle-liver lactylation signaling axis induced by overtraining. Specifically, excessive exercise induces skeletal muscle lactate accumulation, which drives SORBS3 lactylation. This modification promotes the selective sorting of F-box protein 2 (FBXO2) into muscle-derived small extracellular vesicles, resulting in the formation of FBXO2-enriched lactosomes. Upon hepatic uptake, these vesicles directly trigger liver fibrosis (Liu et al., 2025b). This discovery establishes the trans-organ signaling axis of overtraining-driven skeletal muscle SORBS3 lactylation, FBXO2-positive lactosome release, hepatic uptake, and fibrosis induction, providing a mechanistic explanation for the J-shaped relationship between exercise intensity and liver health. It also suggests that muscle-derived lactosomes may be early warning biomarkers for exercise-related liver injury. Currently, these conclusions are primarily based on animal models, and validating the causal relationship between exercise intensity, lactosomes, and liver fibrosis in human populations remains a key future research direction.

### 4.3 Hepatocyte lactylation in fibrotic microenvironment regulation

Beyond HSCs and immune cells, the lactylation status of hepatocytes themselves has received increasing attention for its regulatory role in the fibrotic microenvironment. As the primary lactate producers in the liver, hepatocytes under injury conditions provide lactylation substrate to neighboring cells through enhanced glycolysis, and they also undergo significant changes in their own lactylation state. In hepatic ischemia-reperfusion injury, hepatocyte HSPA12 A suppresses glycolysis-mediated HMGB1 lactylation and secretion, attenuating macrophage chemotaxis and inflammatory injury (Du et al., 2023). In acetaminophen-induced acute liver injury, NEDD4 K33 lactylation activates the Caspase-11-mediated non-canonical pyroptosis pathway to exacerbate liver damage (Li et al., 2024c). These findings suggest that hepatocyte lactylation dictates cell fate (survival versus death) and also reshapes the hepatic immune microenvironment through the release of lactylation-modified damage-associated molecular patterns, constituting a critical link in lactylation signaling among hepatocytes, HSCs, and macrophages. Future research should systematically characterize the dynamic changes in hepatocyte lactylomics at different stages of liver fibrosis to define the independent contribution of hepatocyte lactylation to fibrosis initiation and progression.

### 4.4 The liver-spleen axis in liver fibrosis

The liver and spleen are connected through the portal venous system, and the liver-spleen axis has emerged as an immunometabolic regulatory hub in chronic liver disease (Tarantino and Citro, 2026). Splenic immune remodeling, characterized by altered monocyte and macrophage polarization and elevated pro-inflammatory cytokines such as IL-6 and TNF-alpha, promotes hepatic fibrogenesis through portal venous trafficking of activated immune cells (Dey, 2025). Zhang et al. showed that CD11 b+CD43hiLy6Clo splenocyte-derived macrophages migrate to the liver through the spleno-hepatic circulation and directly exacerbate fibrosis in mice (Zhang et al., 2023). Splenectomy attenuates hepatic fibrogenesis through the LIGHT/TNFSF14/JNK/TGF-beta1 pathway (Liang et al., 2021), and splenic macrophage M2-like polarization modulates portal hypertension-associated cirrhosis (Guihu et al., 2024). These observations indicate that splenic immune remodeling actively contributes to liver fibrosis rather than merely reflecting it. Whether splenic immune cell lactylation participates in this process remains an open question and a promising direction for future study.

## 5 Drug development and therapeutic strategies targeting lactylation

### 5.1 Targeting lactate metabolism and lactylation enzymes

Pharmacological interventions targeting lactylation can be categorized into three hierarchical levels: metabolic substrate supply, modification catalysis, and signal recognition. LDH and HK2 are the two key enzymes governing substrate supply. The LDH inhibitor oxamate suppresses Th17 differentiation and fibrosis progression by reducing lactate levels in arsenic-induced liver fibrosis models (Chen et al., 2025b) and attenuates PRC1 overexpression-exacerbated hepatic steatosis in MAFLD models (Zou et al., 2026) (Table 2). Limonin, as a direct HK2 inhibitor, reduces histone lactylation and inhibits HSC activation (detailed in Section 4.3) (Lan et al., 2026). Upstream metabolic nodes also possess intervention potential: DCA, as a PDHK inhibitor, reactivates pyruvate dehydrogenase to redirect pyruvate metabolism from lactate production to mitochondrial oxidation, reducing H3K18La and inhibiting HSC fibrotic gene expression (Rho et al., 2023). 2-DG suppresses glycolysis to reduce H3K18La levels and inhibit HSC activation (Zhou et al., 2024). Monocarboxylate transporters exhibit bidirectional effects in liver fibrosis. For instance, saikosaponin B1 blocks lactate-driven pro-fibrotic signaling by inhibiting the LDHA-MCT1/4 pathway (Wang et al., 2026b), whereas the MCT1/4 inhibitor syrosingopine paradoxically activates HSCs and exacerbates liver fibrosis (Guo et al., 2024). This suggests that the therapeutic window and direction of MCT intervention may depend on the fibrosis stage and lactate subcellular compartmentalization.

**TABLE 2:** Pharmacological agents and natural products targeting lactylation in liver fibrosis.

Drug/Compound	Development stage	Direct target/Pathway	Mechanism of action in liver fibrosis	Ref
Targeting lactate metabolism and lactylation-modifying enzymes				
Limonin	Preclinical	HK2	Directly binds HK2, inhibits its enzymatic activity, reduces H3K18La, attenuates liver fibrosis *in vivo*	(Lan et al., 2026)
Oxamate	Preclinical	LDH A	Inhibits LDH A, reduces lactate production; suppresses Th17 differentiation but may exacerbate neutrophilic inflammation	(Chen et al., 2025b)
Dichloroacetate (DCA)	Preclinical	PDK	PDK inhibitor; reactivates pyruvate dehydrogenase, promotes mitochondrial oxidation, reduces H3K18La	(Rho et al., 2023)
2-Deoxyglucose (2-DG)	Preclinical	Glycolysis	Inhibits glycolysis, reduces lactate-derived lactylation, suppresses HSC activation	(Zhou et al., 2024)
Curcumol	Preclinical	KAT8	Binds KAT8 (TYR361/THR319), inhibits acetyltransferase activity, blocks HK2-KAT8 interaction	(Guo et al., 2026)
Saikosaponin B1	Preclinical	LDH A/MCT1	Downregulates LDH A and MCT1, reduces lactate production and export, inhibits HSC activation	(Wang et al., 2026b)
Syrosingopine	Preclinical	MCT1/4	MCT1/4 inhibitor; paradoxically activates HSCs and exacerbates fibrosis, indicating context-dependent effects	(Guo et al., 2024)
Ginsenoside Rk1	Preclinical	PFKFB2	Targets PFKFB2, inhibits aerobic glycolysis, blocks HSC epithelial-mesenchymal transition	(Li et al., 2025a)
ACPS-I (polysaccharide)	Preclinical	PKM2	Isolated from Astragalus complanatus seeds; inhibits PKM2, reduces histone lactylation, alleviates fibrosis	(Zhu et al., 2026)
Site-specific lactylation intervention strategies				
PGK1-K353 targeting peptide	Preclinical	PGK1-K353 lactylation	Cell-penetrating peptide blocking PGK1 K353 lactylation in FSP1+ macrophages, delays fibrosis without global lactylation disruption	(Tang et al., 2026)
H4K16La-PDK4 axis	Preclinical	H4K16La/PDK4	H4K16La drives PDK4 expression forming positive feedback in MASLD; PDK4 inhibition reduces lactate and injury	(Jiao et al., 2026)
Myeloid-specific HK2 knockout	Preclinical	HK2 (macrophage-specific)	Myeloid-specific Hk2 deletion breaks HK2-driven H3K18La enrichment in macrophages, reducing fibrosis	(Li et al., 2025b)
Sodium butyrate	Preclinical	HDAC1/H3K18ac	Class I HDAC inhibitor; upregulates H3K18ac to competitively displace H3K18La, validated in HSCs	(Rho et al., 2023)
BRD9	Preclinical	H3K18La reader	H3K18La-specific reader; targeting BRD9 disrupts lactylation-dependent transcription	(Wei et al., 2026)
Natural products and herbal constituents with lactylation-modulating activity				
Limonin	Preclinical	HK2	Most well-studied natural HK2 inhibitor; directly binds HK2, reduces H3K18La, alleviates fibrosis	(Lan et al., 2026)
Salidroside	Preclinical	LDH	Inhibits LDH in overtraining-induced fibrosis model, reduces muscle-derived lactate, alleviates apoptosis and HSC activation	(Liu et al., 2025b)
Huazhuo Tiaozhi Granule	Preclinical	H2B K6/H4 K80 lactylation	TCM formula; promotes hepatocyte H2B K6 and H4 K80 lactylation, inhibits miR-155–5p biogenesis	(Li et al., 2024b)
Salvianolic acid A	Preclinical	BRD4/HMGB1	Inhibits BRD4/HMGB1 pathway, attenuates alcoholic liver injury	(Lan et al., 2020)
Luteolin/Lithospermic acid	Preclinical	FXR	Potent FXR antagonists; targeted metabolic pathways intersect with lactylation networks	(Tu et al., 2025)
ML364	Preclinical	USP2	Inhibits USP2; USP2 K447 lactylation enhances activity, SIRT2 delactylates USP2; anti-fibrotic in multi-organ models	(Han et al., 2025b)

Curcumol acts on the catalysis step, occupying the TYR361/THR319 residues of KAT8 to inhibit its activity and thereby blocking the HK2 K346 lactylation feedback loop, which induces necroptosis of activated HSCs (Guo et al., 2026). Ginsenoside Rk1 targets PFKFB2 to inhibit aerobic glycolysis and block HSC epithelial-mesenchymal transition (Li et al., 2025a). The polysaccharide ACSP-I, isolated from Astragalus complanatus seeds, inhibits PKM2 activity to downregulate MYC and CCND1 expression and reduce histone lactylation, demonstrating anti-hepatic fibrosis potential both *in vitro* and *in vivo* (Zhu et al., 2026). The shared logic of these strategies lies in reducing lactate supply or blocking lactylation catalysis to attenuate H3K18La-driven pro-fibrotic transcriptional programs at their source.

### 5.2 Site-specific intervention strategies for lactylation

Unlike systematic lactylation inhibition, site-specific intervention aims to precisely target pathogenic lactylation events while preserving physiological lactylation functions. Cell-penetrating peptides targeting PGK1-K353 lactylation represent an important advance in this strategy. Distinct from global inhibition of lactate production or broad blockade of lactylation writers, this interfering peptide specifically blocks the single-site modification of PGK1 in FSP1+ macrophages, effectively delaying mouse liver fibrosis progression without disrupting global lactylation homeostasis (Tang et al., 2026) (Table 2). The H4K16La-pyruvate dehydrogenase kinase 4 (PDK4) positive feedback loop drives MAFLD progression, and PDK4 inhibition reduces lactate levels and liver injury (Jiao et al., 2026). In MASH, macrophage HK2-driven H3K18La enrichment at glycolytic gene promoters forms a vicious cycle, and myeloid-specific *HK2* knockout effectively breaks this loop (Li et al., 2025b). Given the direct competition between H3K18La and H3K18ac at the same site, the strategy of using Class I HDAC inhibitors (sodium butyrate) to competitively displace H3K18La by upregulating H3K18ac has been validated in HSCs (Rho et al., 2023). The identification of BRD9 as an H3K18La-specific reader in HCC (Wei et al., 2026) provides a new target for intervention from the signal recognition end, though its role in HSCs remains to be experimentally confirmed. However, current technology cannot yet achieve precise manipulation of the modification status at a single lysine residue, and functional redundancy among different lactylation sites may attenuate the efficacy of single-target strategies. From a trans-organ perspective, H4K16La establishes a positive feedback loop with PDK4 in MAFLD (Jiao et al., 2026), H3K9La drives epithelial cell metabolic reprogramming in intestinal fibrosis (Liu et al., 2025d), and H4K12La regulates nuclear factor-κB (NF-κB) signaling in renal fibrosis (Wang et al., 2024c). This site diversity suggests that future precision interventions should be based on stratified strategies according to fibrosis etiology and stage.

### 5.3 Lactylation regulation by natural products and traditional Chinese medicine compounds

Natural products and active compounds from traditional Chinese medicine hold unique potential for drug discovery in the lactylation regulation field due to their multi-target properties. Limonin is the most extensively studied natural HK2 inhibitor, directly binding to HK2 to inhibit its enzymatic activity and reduce H3K18La levels, significantly alleviating liver fibrosis *in vivo* (Lan et al., 2026) (Table 2). Salidroside reduces skeletal muscle lactate production by inhibiting LDH activity in overtraining-induced liver fibrosis models, thereby decreasing muscle-derived lactosome formation and attenuating hepatocyte apoptosis and HSC activation (Liu et al., 2025b), providing a new candidate molecule for natural anti-fibrotic drug screening targeting LDH. Among compound formulations, Huazhuo Tiaozhi granule promotes hepatocyte H2B K6 and H4 K80 lactylation while inhibiting miR-155–5p biogenesis to exert lipid-lowering effects (Yin et al., 2023), suggesting that traditional Chinese medicine formulations can improve metabolic disorders by remodeling lactylation. Salvianic acid A alleviates alcoholic liver injury by inhibiting the BRD4/HMGB1 pathway (Lan et al., 2020). Luteolin and lithospermic acid have been identified as potent FXR antagonists (Tu et al., 2025), whose target metabolic pathways broadly intersect with the lactylation network. On non-histone proteins, ubiquitin-specific protease 2 (USP2) K447 lactylation is a recently discovered trans-organ fibrosis regulatory hub: TGF-β1-induced USP2 K447La enhances its deubiquitinase activity, while SIRT2 functions as a delactylase to remove this modification and limit USP2 function. The small molecule ML364 inhibits USP2 in renal, hepatic, and pulmonary fibrosis models, demonstrating anti-fibrotic effects across organs (Xiang et al., 2025), which highlights the potential of the lactylation-deubiquitination-ferroptosis axis as a common target for multi-organ fibrosis.

A further obstacle to clinical translation is bioavailability. Limonin is poorly water-soluble and undergoes extensive first-pass metabolism, so plasma concentrations rarely reach the levels needed for anti-fibrotic activity, and preclinical studies also document hepatotoxicity, renal toxicity, and genotoxic potential (Fan et al., 2019; Madanahalli Ramesh et al., 2026). Pharmacokinetic data for other compounds are similarly discouraging. In rats, oral curcumol shows a bioavailability of only 9.2%–13.1% and plasma protein binding of 85.6%–93.4%, leaving little free drug to act on the fibrotic liver (Yang et al., 2022). Most lactylation-modulating natural products have only been tested in cultured cells or rodent models (Agbo et al., 2026). The multi-target activity of these compounds raises additional concern about off-target effects: curcumol and salvianic acid A act on enzymes and pathways well beyond lactylation, and the selectivity of these interactions is largely uncharacterized (Madanahalli Ramesh et al., 2026). Batch-to-batch variability in herbal preparations further complicates quality control and reproducibility (Madanahalli Ramesh et al., 2026). Computational pharmacology, structural derivatization, and delivery technologies may overcome some of these barriers, but the path from promising preclinical data to a clinically useful anti-fibrotic agent remains long (Agbo et al., 2026).

### 5.4 Combination of therapy strategies and clinical translation perspectives

Liver fibrosis involves interactions among metabolic reprogramming, epigenetic remodeling, and immune dysregulation, and single-target intervention against any single aspect of lactylation is unlikely to produce durable benefits. Currently, all lactylation-targeted drugs remain in preclinical research stages, with human efficacy and safety yet to be validated (Liang et al., 2025). Li et al. identified distinct liver fibrosis phenotypes based on lactylation gene signatures, which could guide molecular subtyping-based precision intervention (Li et al., 2024b).

From a strategic perspective, lactylation-targeted therapy can be deployed along three dimensions. The first dimension is metabolic-epigenetic synergistic blockade: simultaneously inhibiting lactate production (HK2/LDH inhibitors) and lactylation writer activity (KAT8/p300 inhibitors) to reduce pathogenic lactylation levels from both substrate supply and modification catalysis ends. The second dimension is epigenetic-immune combination intervention: given that H3K18La-IRF4 signaling drives Th17 differentiation and M2 macrophage polarization, combining LDH inhibitors with immunomodulators may simultaneously block upstream and downstream components of metabolic-immune crosstalk. The third dimension is a lactylation-ubiquitination dual-targeting strategy: based on the competitive regulatory relationship among KAT8, HK2 K346La, and HUWE1-mediated ubiquitination (Guo et al., 2026), designing bifunctional molecules that simultaneously enhance HUWE1 activity and inhibit KAT8 to achieve synergistic regulation of HK2 protein stability. These conceptual frameworks all require rigorous validation in animal experiments and early-phase clinical trials. Before key bottlenecks in lactylation biomarker standardization, drug tissue selectivity optimization, and prospective validation of patient stratification biomarkers are overcome, the clinical translation of lactylation-targeted therapy will remain a stepwise process.

## 6 Future research directions

Although the critical role of lactylation in liver fibrosis has been initially established, the field remains in a phase of rapid development. This review distills priority directions from both mechanistic research and clinical translation perspectives (Figure 3).

**FIGURE 3:**
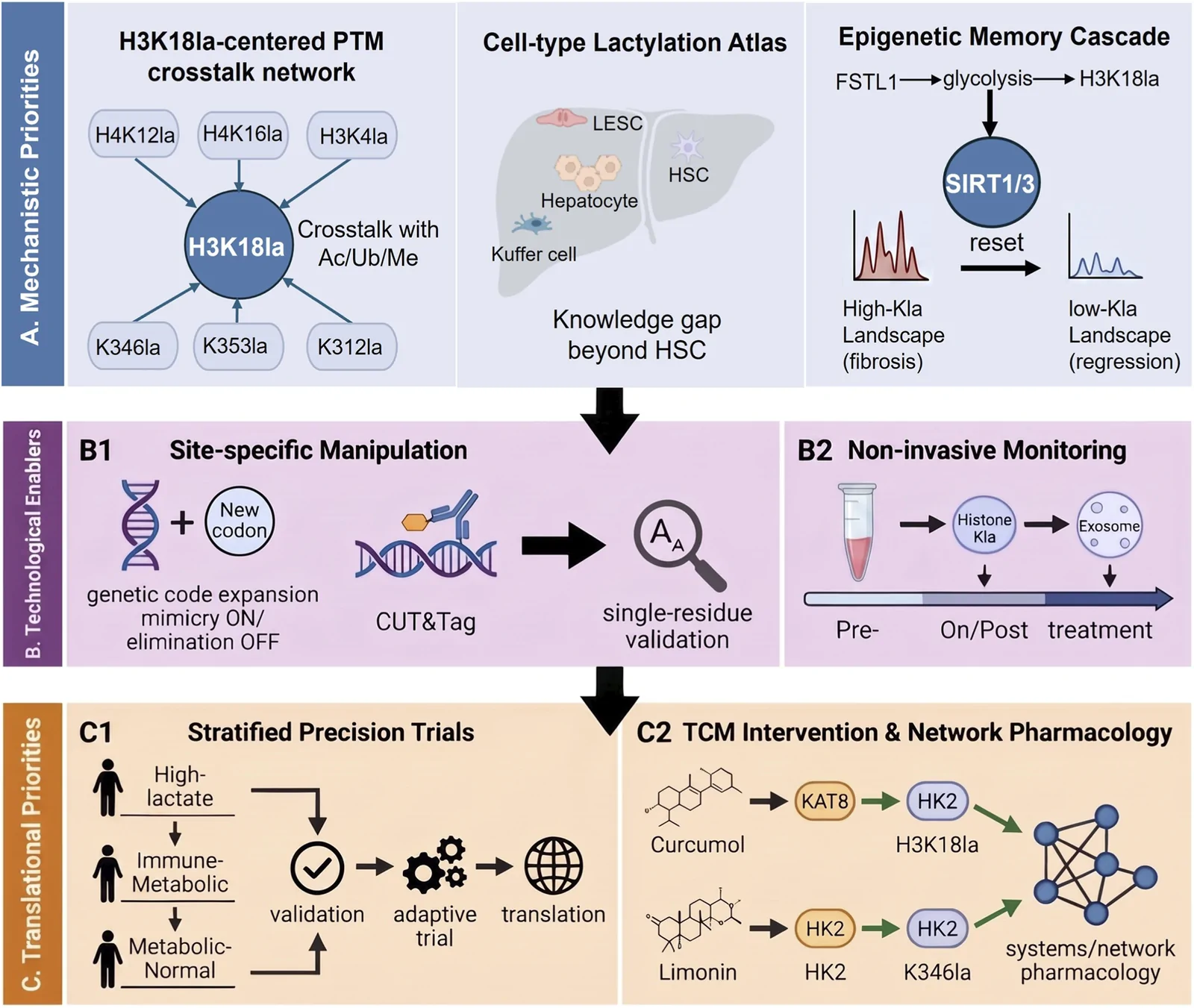
Future research directions and translational priorities for lactylation-targeted anti-fibrotic strategies. Mechanistic priorities **(A)** encompass H3K18La-centered PTM crosstalk networks, cell-type–specific lactylation atlases extending beyond HSCs to Kupffer cells, hepatocytes, and LESCs, and epigenetic memory cascades involving FSTL1-driven glycolysis and SIRT1/3-mediated landscape resetting. Technological enablers **(B)** include site-specific genetic code expansion, CUT&Tag, single-residue validation, and non-invasive histone lactylation monitoring via circulating exosomes. Translational priorities **(C)** comprise stratified precision trials based on lactate–immune–metabolic subtypes and traditional Chinese medicine network pharmacology targeting the HK2-KAT8 and HKDC1-H3K18La axes. Abbreviations: FSTL1, follistatin-like protein 1; H3K18La, histone H3 lysine 18 lactylation; HK2, hexokinase 2; HKDC1, hexokinase domain-containing protein 1; KAT8, lysine acetyltransferase 8; Kla, histone lysine lactylation; LSECs, liver sinusoidal endothelial cells; PTM, post-translational modification; SIRT1/3, sirtuin 1/3; TCM, traditional Chinese medicine.

### 6.1 Mechanistic research priorities

Four mechanistic questions stand out as priorities. First, the multi-site PTM cooperative regulatory network centered on H3K18La. This review focuses on the crosstalk between H3K18La and acetylation, ubiquitination, and methylation. However, emerging evidence—including the coupling of H4K12La with NF-κB (Wang et al., 2024c), the metabolic positive feedback of H4K16La with PDK4 (Jiao et al., 2026), the non-histone HK2 K346La-ubiquitination seesaw (Guo et al., 2026), PGK1 K353La-phosphorylation cooperation (Tang et al., 2026), and ASH2L K312La-mediated indirect remodeling of H3K4 methylation (demonstrated in HCC models (Han et al., 2025b))—suggests that the lactylation PTM interaction network extends far beyond a single node. Systematically mapping the lactylation-PTM interactome in fibrosis will be a key step toward understanding metabolic-epigenetic signal integration mechanisms. Second, cell type-specific lactylation atlases of the liver fibrosis microenvironment. Current research primarily focuses on HSCs, while the lactylation characteristics of hepatocytes, liver sinusoidal endothelial cells, and different immune subsets during fibrogenesis remain unexplored. Systematic characterization of lactylation heterogeneity within the fibrotic niche using Kupffer cell global lactylome analysis (Sung et al., 2023) and lactylproteomics technologies (Ran et al., 2025) will help identify cell type-specific therapeutic targets. Third, validation of lactylation as fibrotic epigenetic memory. The discovery that FSTL1 drives cartilage fibrosis through glycolysis-dependent H3K18La (Lu et al., 2026) raises the important hypothesis that lactylation may maintain fibrotic predisposition at the metabolic-epigenetic level. Elucidating how delactylases such as SIRT1/3 reset lactylation during fibrosis resolution will determine whether lactylation is a reversible target for preventing fibrosis recurrence. Fourth, splenic lactylation and the liver-spleen axis. Whether splenic immune cell lactylation contributes to liver fibrosis has not been tested directly. Activated macrophages and monocytes generate lactate through aerobic glycolysis, providing substrate for lactylation within the splenic microenvironment (Dey, 2025; Zhang et al., 2023), and the portal circulation could deliver spleen-derived lactate and lactylation-modified damage-associated molecular patterns directly to the hepatic sinusoids (Yoshida et al., 2023). These proposed mechanisms remain speculative and await profiling of the splenic lactylome in fibrosis models, together with spleen-specific genetic tools and spleen-to-liver cell tracking.

### 6.2 Translational and technological advances

To facilitate clinical translation, four technical developments are needed before lactylation can move toward the clinic. First, development of site-specific lactylation manipulation tools. Site-specific lactylation mimicry and erasure based on genetic code expansion technology (Han et al., 2025b), combined with high-resolution techniques such as CUT&Tag (Ran et al., 2025), will enable causal validation of individual lysine residue functions, advancing the field from associative description to mechanistic dissection. Second, establishment of non-invasive dynamic lactylation monitoring systems. There is an urgent need to develop liquid biopsy technologies based on circulating histone lactylation and exosomal lactylated proteins to achieve longitudinal assessment of lactylation status in fibrosis patients. Third, lactylation subtyping-based precision clinical trials. The lactylation-based stratification framework proposed by Li et al. (Li et al., 2024b) requires prospective cohort validation of its predictive value and the design of biomarker-driven adaptive trials to accelerate clinical translation of lactylation-targeted therapy. Finally, lactylation intervention from the perspective of integrated Chinese and Western medicine. The unique advantages of multi-target lactylation regulation by traditional Chinese medicine (e.g., curcumol targeting KAT8, limonin targeting HK2) warrant systematic and network pharmacology approaches to elucidate the molecular mechanisms underlying their synergistic regulation of the lactylation network. Such studies would provide scientific evidence for developing novel anti-fibrotic traditional Chinese medicines.

## 7 Conclusion

The discovery of lactylation has redefined lactate from a metabolic waste product to a key messenger linking metabolic dysregulation with epigenetic regulation. In liver fibrosis, HK2, HKDC1, and IGF2BP2 are proposed to converge on H3K18La through distinct metabolic pathways to drive pro-fibrotic transcriptional programs. This convergence hypothesis constitutes the core theoretical framework of this review, while the independent roles of H4K12La and H4K16La in metabolic liver disease suggest that a multi-node coordination model also warrants consideration. From a drug development perspective, strategies such as using limonin to target HK2, curcumol to inhibit KAT8, salidroside to block LDH, and PGK1 site-specific interfering peptides have demonstrated the potential of lactylation as a therapeutic target at the three levels of metabolic substrate, modification catalysis, and site recognition, though all drugs remain in preclinical stages. The field is currently constrained by several critical bottlenecks, including insufficient assay standardization, limited drug selectivity, and the translation gap from animal models to humans. Looking forward, single-cell lactylomics technologies, mapping of lactylation-PTM interactomes, and molecular subtyping-based clinical trial design will drive the transformation of lactylation from basic discovery toward liver fibrosis diagnosis and treatment.
